# Organic Nitrogen Uptake and Assimilation in *Cucumis sativus* Using Position-Specific Labeling and Compound-Specific Isotope Analysis

**DOI:** 10.3389/fpls.2018.01596

**Published:** 2018-11-06

**Authors:** Pierre-Paul Dion, Sandra Jämtgård, Annick Bertrand, Steeve Pepin, Martine Dorais

**Affiliations:** ^1^Department of Plant Sciences, Centre de recherche et d’innovation sur les végétaux, Université Laval, Québec, QC, Canada; ^2^Department of Forest Ecology and Management, Swedish University of Agricultural Sciences, Umeå, Sweden; ^3^Agriculture and Agri-Food Canada, Quebec Research and Development Centre, Québec, QC, Canada; ^4^Department of Soil and Agri-Food Engineering, Centre de recherche et d’innovation sur les végétaux, Université Laval, Québec, QC, Canada

**Keywords:** amino acid, assimilation, compound-specific stable isotope analysis, cucumber, nitrogen, organic horticulture, position-specific labeling, uptake

## Abstract

Organic nitrogen is now considered a significant source of N for plants. Although organic management practices increase soil organic C and N content, the importance of organic N as a source of crop N under organic farming management systems is still poorly understood. While dual-labeled (^13^C and ^15^N) molecule methods have been developed to study amino acid uptake by plants, multiple biases may arise from pre-uptake mineralization by microorganisms or post-uptake metabolism by the plant. We propose the combination of different isotopic analysis methods with molecule isotopologues as a novel approach to improve the accuracy of measured amino acid uptake rates in the total N budget of cucumber seedlings and provide a better characterization of post-uptake metabolism. Cucumber seedlings were exposed to solutions containing L-Ala-1-^13^C,^15^N or U-L-Ala-^13^C_3_,^15^N, in combination with ammonium nitrate, at total N concentrations ranging from 0 to 15 mM N and at inorganic/organic N ratios from 10:1 to 500:1. Roots and shoots were then subjected to bulk stable isotope analysis (BSIA) by Isotope Ratio Mass Spectrometry (IRMS), and to compound-specific stable isotope analysis (CSIA) of the free amino acids by Gas Chromatography – Combustion – Isotope Ratio Mass Spectrometry (GC-C-IRMS). Plants exposed to a lower inorganic:organic N ratio acquired up to 6.84% of their N from alanine, compared with 0.94% at higher ratio. No ^13^C from L-Ala-1-^13^C,^15^N was found in shoot tissues suggesting that post-uptake metabolism of Ala leads to the loss of the carboxyl-C as CO_2_. CSIA of the free amino acids in roots confirmed that intact Ala is indeed taken up by the roots, but that it is rapidly metabolized. C atoms other than from the carboxyl group and amino-N from Ala are assimilated in other amino acids, predominantly Glu, Gln, Asp, and Asn. Uptake rates reported by CSIA of the free amino acids are nevertheless much lower (16–64 times) than those reported by BSIA. Combining the use of isotopologues of amino acids with compound-specific isotope analysis helps reduce the bias in the assessment of organic N uptake and improves the understanding of organic N assimilation especially in the context of organic horticulture.

## Introduction

Nitrogen is usually considered as being more readily available for plant uptake in its inorganic forms, NO_3_^-^ and NH_4_^+^ (Nacry et al., 2013). However, it is now well established that plants can also take up N directly as organic molecules (Näsholm et al., 2009) such as amino acids and peptides, as most of the transporters and related genes are known, at least for *Arabidopsis thaliana* (Rentsch et al., 2007; Tegeder and Rentsch, 2010). The capacity to use organic N allows plants to access a wide array of N sources (Paungfoo-Lonhienne et al., 2012; Warren, 2014), and thus it is important to consider the availability of organic molecules in fertilization planning in agriculture.

The uptake of organic N (ON) was, at first, considered a secondary source of N, mostly useful in environments with low inorganic N (IN) availability such as arctic or boreal ecosystems (Näsholm et al., 1998; Henry and Jefferies, 2003; Schimel and Bennett, 2004; Zhou et al., 2013; Hu et al., 2017). Conversely, more recent studies on agricultural (Näsholm et al., 2000; Okamoto and Okada, 2004; Jämtgård et al., 2008; Ge et al., 2009; Gioseffi et al., 2012; Brackin et al., 2015; Moran-Zuloaga et al., 2015) and sub-tropical plants (Wei et al., 2015) demonstrated that ON uptake accounts for a significant portion of the N budget of the plant, although IN is still often the preferred N source. In addition, organic molecules have been found to be the preferred N source for some tree species, even when IN is abundant (e.g., beech, see Li et al., 2015). Organic N molecules have the advantage of providing reduced N along with a C source, which could improve the C budget and N use efficiency of the plant by minimizing the energy required for N reduction and assimilation (Franklin et al., 2017; Ganeteg et al., 2017). It has also been established that plants can take up ON even in the presence of IN (Gioseffi et al., 2012; Gruffman et al., 2014; Czaban et al., 2016), sometimes with no effect on ON uptake rate (e.g., in wheat, Gioseffi et al., 2012). Nonetheless, knowledge of the effect of varying availability of IN relative to ON (IN:ON ratio) on the uptake of ON, at varying total N concentrations, is still missing and could help determine the nutritional contexts in which ON uptake is most relevant.

Organic agriculture is increasingly considered as a realistic and more sustainable alternative to conventional intensive agriculture for meeting the global challenges in food safety and security (Reganold and Wachter, 2016). In order to meet the requirements for organic certification, N must be provided to the crops entirely or predominantly as organic residues. Specifically, greenhouse crops require very high levels of fertilization (Cuijpers et al., 2008; Wang et al., 2017; Dorais and Schwarz, 2018). The synchronization of N supply by organic fertilizers with N demand by the crop was identified as one of the most important challenges in the next years for improving yields, while reducing environmental impacts of greenhouse organic horticulture (Reeve et al., 2016; Tittarelli et al., 2016). Organic management of soils generally increases the total organic C (Ge et al., 2011) and the concentration of free amino acids in the soil solution (Ge et al., 2012). Paradoxically, the uptake of organic N molecules has rarely been studied in open field organic horticulture (Song et al., 2010), and never in greenhouse organic horticulture where applications of organic amendments are frequent. Better knowledge of the significance of the uptake of organic N molecules in the global N budget of organic horticultural plants, especially in greenhouses, could help improving the planning of organic fertilization.

The use of dual-labeled (^13^C and ^15^N) organic molecules combined with bulk stable isotope analysis (BSIA) is one of the key methods used for ON root uptake experiments. Monitoring the ^13^C intake along with ^15^N, following exposure of the roots to a dual-labeled amino acid, increases the certainty that the isotopic enrichment is due to intact amino acid uptake, rather than uptake of IN following mineralization of the labeled amino acid. Universally labeled molecules (UL), in which all the C and N atoms are of the heavy isotope type, are typically used for ON root uptake experiments (e.g., Näsholm et al., 1998; Ge et al., 2009; Czaban et al., 2016; Franklin et al., 2017). They are available at a reasonable cost and have the advantage of providing a strong isotopic signal in the plant. The monitoring of ON uptake with BSIA can be biased by pre-uptake metabolism of the intact molecule by soil microbes or by post-uptake metabolism (Sauheitl et al., 2009; Rasmussen et al., 2010) and ^13^CO_2_ respiration inside the plant (Ganeteg et al., 2017). Compound specific isotope analysis (CSIA) by gas chromatography – combustion – isotope-ratio mass spectrometry (GC-C-IRMS) has recently been used in the study of amino acid uptake to reduce the bias associated with the use of BSIA, by monitoring the isotopic signature specifically in amino acids of interest (Sauheitl et al., 2009). Analysis of the intact universally labeled amino acid using liquid chromatography – mass spectrometry (LC-MS) is another CSIA method for detection of the intact amino acid molecule taken up by the plant (Gaudin et al., 2014; Czaban et al., 2016; Ganeteg et al., 2017). Due to the rapid metabolism of amino acids following their uptake, these methods are not free from biases in the evaluation of intact ON uptake and for these reasons underestimate uptake (Ganeteg et al., 2017). It is nevertheless a tool with great potential for determining uptake rates and for studying the post-uptake metabolism of ON and its related C inside the plant. Using all of these tools, the duration of the exposure of the plants to the labeled solution and their subsequent processing must be planned with care due to the fast processes of degradation in the soil (or nutrient solution) and plant metabolism after uptake. Moran-Zuloaga et al. (2015) compared the use of UL and position-specific labeled (PSL) amino acids and reported that UL amino acids overestimate the uptake of intact molecules measured by BSIA, relative to PSL amino acids. They suggest using amino acids with only the carboxyl-C atom as ^13^C, because this C is usually the first to be removed from the molecule by decarboxylation from the action of soil microorganisms or plant metabolism. Thus, the use of PSL amino acids may provide more accurate estimates of uptake rates of intact amino acids compared to UL amino acids. More specifically, using CSIA in an experiment combining the use of UL and PSL molecules would provide information on the fate of specific C atoms from the labeled amino acid taken up by the plant.

The objective of the present study was to characterize the significance of organic N acquisition as alanine in the total N budget of cucumber seedlings grown under different N forms and concentrations, and IN:ON ratios relevant to greenhouse organic horticulture. The underlying hypothesis was that cucumber plants rely mostly on inorganic N as N source but, when exposed to low IN:ON ratios or low total N availability, organic N source could become significant. To discriminate between the N sources utilized by the plants, we used dual labeled Alanine (^13^C and ^15^N) as source of organic N. We also wanted to test the combined approach of using CSIA with PSL amino acids to better understand the fate of amino acids taken up within the cucumber plant following exposure to labeled Alanine.

## Materials and Methods

### Experimental Setup and Labeled Solutions

The experiment was set up as five complete randomized blocks, with 19 plants per block. Each plant was exposed to one of 19 different labeled solutions for a period of 2 h. The labeled solutions were a mixture of dual-labeled L-Alanine (either L-Ala-1-^13^C,^15^N with ^13^C at the carboxyl-C, or universally labeled L-Ala-^13^C_3_,^15^N; purchased from Sigma-Aldrich) as ON source, and unlabeled ammonium nitrate (NH_4_NO_3_) as IN source.

The contents of labeled solutions are detailed in Table 1. The maximum N concentration (15 mM) is the suggested N concentration in fertigation for conventional greenhouse horticulture of cucumber in Ontario, Canada (OMAFRA, 2010). Solutions S1–S9 consist of a combination of three IN:ON ratios, at three different total N concentrations, using Ala-1-^13^C,^15^N as the ON source, to test how varying N concentration and IN:ON ratio affect Ala uptake. Solutions S7–S18 aimed at comparing the use of UL and PSL Ala, in presence or absence of IN. Solutions S13–S18 contain U-Ala-^13^C_3_,^15^N as the ON source. Solutions S13–S15 are the same as S7–S9 (IN:ON ratio of 10:1, at three different total N concentrations), but with the different types of labeled Ala. Solutions S10–S12 and S16–S18 contain only amino acids, without ammonium nitrate, at the same Ala concentrations as, respectively, solutions S7–S9 and S13–S15. CSIA was performed on free amino acids in roots and shoots from plants exposed to solutions S8, S11, S14, S17, and S0.

**Table 1:** Composition of the 19 labeled solutions to which the cucumber seedlings were exposed.

Solution #	Total N (mM N)	IN:ON ratio (mM N)	Labeled Ala	
0	0	—	—	
1	0.5	500:1	Ala-1-^13^C,^15^N	PSL
2	5	500:1	Ala-1-^13^C,^15^N	
3	15	500:1	Ala-1-^13^C,^15^N	
4	0.5	100:1	Ala-1-^13^C,^15^N	
5	5	100:1	Ala-1-^13^C,^15^N	
6	15	100:1	Ala-1-^13^C,^15^N	
7	0.5	10:1	Ala-1-^13^C,^15^N	
8	5	10:1	Ala-1-^13^C,^15^N	
9	15	10:1	Ala-1-^13^C,^15^N	
10	0.045^∗^	100% ON	Ala-1-^13^C,^15^N	
11	0.455	100% ON	Ala-1-^13^C,^15^N	
12	1.364	100% ON	Ala-1-^13^C,^15^N	
13	0.5	10:1	U-Ala-^13^C_3_,^15^N	UL
14	5	10:1	U-Ala-^13^C_3_,^15^N	
15	15	10:1	U-Ala-^13^C_3_,^15^N	
16	0.045^∗^	100% ON	U-Ala-^13^C_3_,^15^N	
17	0.455	100% ON	U-Ala-^13^C_3_,^15^N	
18	1.364	100% ON	U-Ala-^13^C_3_,^15^N	

*Inorganic N (IN) was supplied as NH_*4*_NO_*3*_, and organic N (ON) as either Ala-1-^*13*^C,^*15*^N or U-Ala-^*13*^C_*3*_,^*15*^N. Plants exposed to the solutions in gray were also analyzed by CSIA, in addition to BSIA. *^∗^Solutions 10–12 and 16–18 had the same molar concentration of Ala as, respectively, solutions 7 to 9 and 13–15. The difference in total N comes from the absence of NH_*4*_NO_*3*_. For all treatments, n = 5, except S1, 2, 3, 4, 5, 6, 15, and 18, for which n = 4.**

We used ammonium nitrate as IN source to avoid the possible interaction between N uptake and the presence of another cation. According to Roosta and Schjoerring (2007), cucumber growth is not hindered by ammonium toxicity when ammonium is provided in equimolar concentration with nitrate. The choice of Alanine (Ala) as organic N source was based on the results of a previous greenhouse experiment on cucumber in which organic N amendments were provided by a mixture of blood meal and feather meal in a peat moss-based organic substrate (Dion et al., 2017). The analysis of cucumber xylem sap samples and soil water extracts showed that Ala, which was the most abundant amino acid in the substrate, was also present in the xylem sap showing its ability to be taken up by cucumber. The 10:1, 100:1, and 500:1 IN:ON ratios are approximations of the ratios of inorganic-N to, respectively, soluble organic-N, amino acid-N and Ala-N in the soil that were observed in the same experiment.

### Plant Growth and Sampling

A total of 116 seeds of cucumber (*Cucumis sativus* cv. Verdun) were placed between two layers of wet brown paper in 100 × 20 mm petri dishes (two seeds per dish) and let at room temperature for germination. The paper was kept wet daily by the addition of deionized water. After 4 days, when the cotyledons started to unfold, the seedlings, still in their brown paper to support the roots, were transferred in 50 mL tubes (one seedling per tube), with a few mL of deionized water to keep the paper moist. The tubes were placed in a growth chamber for 1 h the first day, 2 h the second day, 10 h the third day, and the entire day henceforth, to acclimate the plants to growth chamber conditions. The growth chamber provided the following conditions: 25°C temperature, relative humidity of 75% and 750 μmol ⋅ m^-2^ ⋅s^-1^ PPFD at leaf height, with a photoperiod of 18 h. Four days after their transfer into 50 mL tubes, when the cotyledons were fully expanded and the first leaf started to unfold, damaged seedlings were discarded and 19 healthy seedlings per complete randomized block, for a total of 95 plants, were transferred in 250 mL Mason jars (one plant per jar) filled with the following nutritive solution: 0.85 g ⋅ L^-1^ calcium nitrate, 1.15 g ⋅ L^-1^ of 6-11-31 (N-P_2_O_5_-K_2_O) hydroponic fertilizer (Plant-Prod, Brampton, ON, Canada) and 0.013 g ⋅ L^-1^ L-Alanine. The resulting mineral content of the growth solution was (mg ⋅ L^-1^): 203 N (IN:ON ratio 100:1), 55.2 P, 296 K, 34.5 Mg, 208 Ca, 40 S, 3.45 Fe, 0.69 Mn, 0.23 Zn, 0.0046 Cu, and 0.31 B.

In order to limit size difference among plants at the time of sampling, the seeds were separated in two groups: the first one for blocks 1–3, and the second one for blocks 4 and 5. The seeds of blocks 4 and 5 were humidified 3 days after those of blocks 1–3, and all following manipulations were also delayed 3 days relative to blocks 1–3.

Exposure to the labeled solution was performed when the plants in the first block had two true leaves fully unfolded. One complete block was sampled each day, for 5 consecutive days. The plants were sampled in sequence from the lowest to the highest ^13^C and ^15^N concentrations in order to limit contamination. The roots of each plant were submerged once in a 5 mM CaCl_2_ solution and twice in deionized water (the solution and the deionized water were replaced after each plant), then placed in a new 250 mL Mason jar containing 195 mL of the labeled solution. The plants were exposed 2 h to the solution, then sampled. The roots and the shoot were separated. The roots were again submerged once in CaCl_2_ 5 mM and twice in deionized water. The shoot was also washed with deionized water in order to remove potential fertilizer residues. The volume of the remaining (not taken up by the plant) labeled solution was measured in a graduated cylinder. Fifteen milliliter of this solution was sampled and frozen for subsequent NO_3_^-^ and NH_4_^+^ analysis by ion chromatography (ICS-1100 for NH_4_^+^ and ICS-2100 for NO_3_^-^; Dionex Corporation, Sunnyvale, CA, United States) for calculating the IN uptake. The clean roots and shoots were placed separately in pre-weighted Al envelopes and immediately frozen in liquid N. They were then kept at -80°C for a few days before being freeze-dried, weighed and ground to powder in a mortar with pestle.

### Isotopic Analyses

Dried and ground samples were enclosed in tin capsules and sent to the Stable Isotope Facility at the University of California, Davis, CA, United States, for BSIA of the ^13^C and ^15^N enrichment along with total C and N content (PDZ Europa ANCA-GLS elemental analyzer interfaced to a PDZ Europa 20-20 isotope ratio mass spectrometer; Sercon Ltd., Crewe, Cheshire, United Kingdom).

Based on the method described by Walsh et al. (2014), the remaining of samples exposed to solutions S0, S8, S11, S14, and S17 were used for CSIA of ^13^C and ^15^N in free amino acids by gas chromatography – combustion – isotope ratio mass spectrometry (GC-C-IRMS), in a Trace GC Ultra gas chromatograph, coupled to a Thermo Delta V Plus isotope ratio mass spectrometer through a GC IsoLink (Thermo Scientific, Waltham, MA, United States) at the Stable Isotope Facility at the University of California. Free amino acids were extracted in ethanol 80% for 30 min. Analyzed amino acids were Ala, Asx, Glx, Gly, Ile, Leu, Lys, Met, Phe, Pro, Thr, Tyr, and Val. Gln and Glu are grouped as “Glx” and Asn and Asp are grouped as “Asx,” because the columns used did not allow complete separation of the corresponding peaks. Lys, Met, Tyr, and Thr were below the limit of quantification in all samples.

#### Quantification of Free Amino Acids

Plant samples exposed to solutions S0, S8, S11, S14, and S17 yielded just enough dry material for CSIA. Since GC-C-IRMS does not allow quantification of the amino acids enriched in ^13^C or ^15^N, free amino acids were quantified in other samples and extrapolated to the plants submitted to CSIA. Free amino acids from the freeze-dried and ground samples exposed to solutions S1, S5, S7, S9, S10, and S12 were extracted by suspending 0.2 g leaves and 0.1 g roots in, respectively, 7 and 4 mL 80% ethanol (Carpena-Ruiz et al., 1989). The ethanol extracts were evaporated, then the residue containing the amino acids was re-solubilized in water. The amino acids were derivatized using the AccQ⋅Tag^TM^ Ultra method, before quantification in a UPLC (Waters Corporation, Milford, MA, United States). The concentrations of free amino acids in samples that were quantified are presented in Supplementary Figure 1. The correspondence between samples in which free amino acids were quantified (S1, S5, S7, S9, S10, and S12) and samples submitted to CSIA (S0, S8, S11, S14, and S17) is detailed in Supplementary Table 1.

#### Uptake Calculations

The ^13^C and ^15^N excess in root and shoot samples were calculated using the difference in atom% between samples exposed to the labeled and unlabeled (S0 in Table 1) solutions and converted to μmol (^15^N or eq-^13^C) ⋅ g^-1^ dw ⋅ h^-1^ using the dry weight and total C or N content of the samples. In order to allow a comparison of the assessment of ^13^C enrichment by both types of labeled Ala (Ala-1-^13^C,^15^N or U-Ala-^13^C_3_,^15^N), the excess in μmol equivalent ^13^C (“eq-^13^C”) was estimated by dividing by 3 the ^13^C excess in samples exposed to U-Ala-^13^C_3_,^15^N. The assimilation of ^13^C and ^15^N in free amino acids was expressed in nmol (^15^N or eq-^13^C) ⋅ g^-1^ dw ⋅ h^-1^, by multiplying the free amino acid ^13^C or ^15^N enrichment (in nmol ^15^N or eq-^13^C ⋅ mol^-1^ AA ⋅ h^-1^) by the free amino acid concentration (mol AA ⋅ g^-1^ dw).

The IN uptake was calculated using the NO_3_^-^ and NH_4_^+^ depletion in the labeled solution and the volume of solution absorbed by the roots. This allowed to express the Ala uptake as mmol eq-^13^C or ^15^N ⋅ mol^-1^ total N, in order to study the proportion of total N uptake due to Ala uptake.

### Statistical Analyses

Statistical analyses were performed using *R* 3.4.3 (R Core Team, 2018) with packages *aod* 1.3 (Lesnoff and Lancelot, 2012; for Wald test), *lme4* 1.1–15 (Bates et al., 2015), *lmerTest* 2.0–36 (Kuznetsova et al., 2017), and *multcomp* 1.4–8 (Hothorn et al., 2008).

An ANOVA followed by a Dunnett test (α = 0.05) was first performed comparing the bulk ^13^C and ^15^N enrichments of shoots and roots of the plants exposed to all labeled solutions (S1–S18) with those exposed to the non-labeled control solution (S0).

A non-linear regression following the Michaelis-Menten kinetics was performed on the eq-^13^C and the ^15^N rate of enrichment (*v*) against the Ala-N concentration [Ala] of the solution to which the plant was exposed, in order to estimate the maximum uptake rate, *K_m_*, and the affinity constant, *V_max_*, parameters.

$$\begin{array}{c} v=\frac{V_{max}[Ala]}{K_{m}+[Ala]} \end{array}$$

In all curves, the eq-^13^C or ^15^N enrichment of plants exposed to S0 was used for 0 mM Ala. The Wald test was used to perform pairwise comparisons of the parameters among treatments. The *P*-values were adjusted according to the Benjamini–Hocheberg procedure with Q = 0.05.

Given the high number of different treatment solutions, they were separated in two sub-experiments in regard to the statistical analyses. K_m_ and V_max_ parameters were compared among the three curves given by the different IN:ON ratios with Ala-1-^13^C,^15^N (solutions S1–S9 in Table 1) to test how varying N concentration and IN:ON ratio affect Ala uptake. They were also compared among the four curves resulting from the use of the two types of labeled Ala, with and without IN (solutions S7–S18), to compare the use of UL and PSL Ala, in presence or absence of IN.

As a complementary data analysis, a two-way and a three-way ANOVAs (α = 0.05) with mixed-effect models were performed to test the effects of total N concentration, the IN:ON ratio and their interaction (solutions S1–S9) and the effects of the type of isotopic labeling, the Ala concentration, the presence/absence of NH_4_NO_3_ and their interactions (solutions S7–S18) on the eq-^13^C or ^15^N enrichment in roots and shoots. Blocks were considered to be a random effect. The results are presented in Supplementary Tables 3, 4.

An ANOVA of the effects of the kind of labeled molecule (I), the presence or absence of IN (N) and their interaction (I × N) on the eq-^13^C and ^15^N enrichment of the free amino acids was performed (α = 0.05) for each amino acid, among the four treatments concerned (S8, 11, 14, and 17). Blocks were included as a random effect.

## Results

**FIGURE 1:**
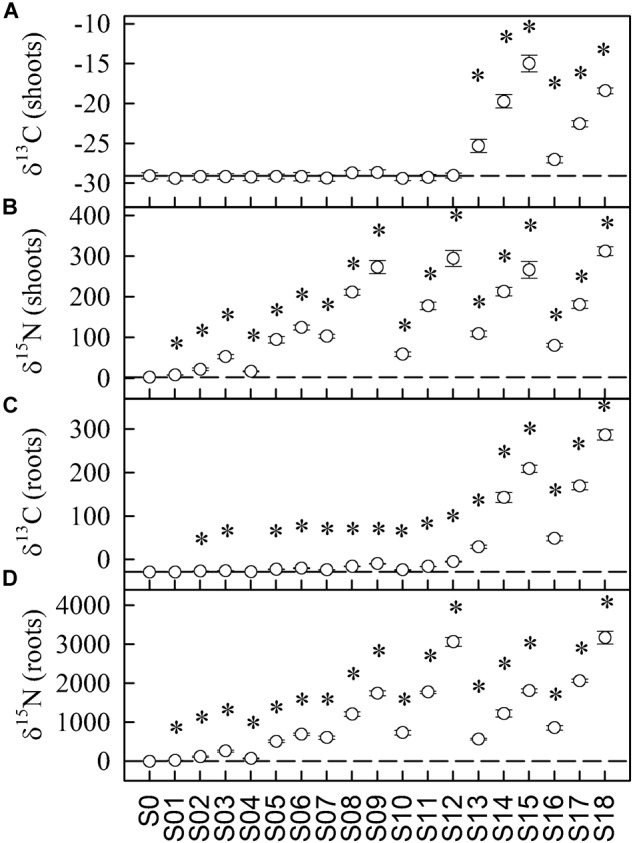
^13^C **(A,C)** and ^15^N **(B,D)** content (expressed as δ values obtained by BSIA) of shoots **(A,B)** and roots **(C,D)** of cucumber seedlings exposed to 19 different labeled solutions. See Table 1 for the detailed content of the solutions. Stars indicate that the shoots or roots of the plants exposed to these solutions were significantly enriched in ^13^C or ^15^N relative to the unlabeled control solution (SO), according to the Dunnett test (α = 0.05). Values are means ± SE. Horizontal dashed lines are the δ^13^C and δ^15^N values of plants exposed to the unlabeled control solution. For all treatments, *n* = 5, except S1, 2, 3, 4, 5, 6, 15, and 18, for which *n* = 4.

Both the shoots and roots of plants were significantly enriched in ^15^N in all treatments involving Ala-1-^13^C,^15^N (Figures 1B,D; solutions S1–S12). Furthermore, some ^13^C did enter the roots in most of those treatments (Figure 1C) but did not reach the shoots (Figure 1A). In response to plant exposure to solutions containing U-Ala-^13^C_3_,^15^N, we observed that ^13^C and ^15^N reached cucumber shoots and roots at all concentrations (Figure 1; solutions S13–S18). The linear regressions of ^13^C enrichment against ^15^N enrichment (following method of uptake calculation from Näsholm et al., 1998) within each treatment are presented in Supplementary Figure 2 and Supplementary Table 2.

**FIGURE 2:**
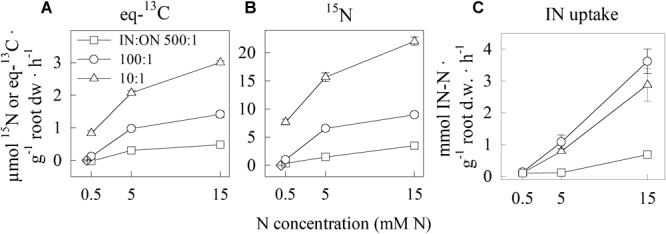
Influence of total N concentration and IN:ON ratio on eq-^13^C **(A)** and ^15^N **(B)** enrichment in whole plants of cucumber seedlings per root dry weight based on BSIA, and on the IN uptake by the plant **(C)** based on IN depletion in the solution. Plants were exposed to nine different solutions (S1–S9 in Table 1) of three total N concentrations × three different IN:ON ratios (IN = NH_4_NO_3_-N and ON = Alanine-1-^13^C,^15^N-N): 500:1 (□), 100:1 (∘) and 10:1 (∆). Diamonds (◇) are enrichment at 0 mM N. Values are mean ± SE. *n* = 4 for each ratio in plants exposed to 0.5 and 5 mM N; *n* = 5 for the others.

The uptake of Ala followed Michaelis-Menten kinetics correspondingly to the concentration of Ala to which the roots were exposed. In a solution with a lower IN:ON ratio, the cucumber plants had an affinity constant (K_m_) and a maximum uptake rate (V_max_) significantly higher than when exposed to a higher IN:ON ratio (Table 2A and Figures 2A,B). IN uptake increased by 25 and 37% when raising the IN:ON ratio from 10:1 to 100:1, but decreased five and nine-fold at a IN:ON ratio of 500:1, when exposed to a total N concentration of, respectively, 5 and 15 mM (Figure 2C).

**Table 2:** K_m_ and V_max_ parameters of eq-^13^C and ^15^N uptake from labeled Alanine in cucumber seedlings.

A			eq-^13^C		^15^N	
Plant part	IN:ON ratio	*n*	K_m_ ± SE (mM Ala-N)	V_max_ ± SE (μmol eq-^13^C ⋅ g^-1^ dw ⋅ h^-1^)	K_m_ ± SE (mM Ala-N)	V_max_ ± SE (μmol ^15^N ⋅ g^-1^ dw ⋅ h^-1^)
Whole plant	10:1	20	0.19 ± 0.04 a	3.27 ± 0.16 a	0.10 ± 0.02 a	22.02 ± 0.74 a
uptake	100:1	17	0.05 ± 0.01 b	1.87 ± 0.17 b	0.04 ± 0.02 b	11.33 ± 1.80 b
per root d.w.	500:1	17	0.02 ± 0.01 b	0.83 ± 0.20 b	0.06 ± 0.15 ab	10.3 ± 18.5 ab

**B** **Plant part**	**Trt**		**K_m_ ± SE** **(mM Ala-N)**	**V_max_ ± SE** **(μmol eq-^13^C ⋅ g^-1^ dw ⋅ h^-1^)**	**K_m_ ± SE** **(mM Ala-N)**	**V_max_ ± SE** **(μmol ^15^N ⋅** **g^-1^ dw ⋅ h^-1^)**

Shoots	PSL + IN	20			0.08 ± 0.01 b	1.39 ± 0.05 b
	PSL	20			0.52 ± 0.14 a	2.03 ± 0.21 a
	UL + IN	19	0.22 ± 0.06	0.94 ± 0.07	0.07 ± 0.02 b	1.34 ± 0.07 b
	UL	19	0.42 ± 0.16	0.79 ± 0.11	0.45 ± 0.14 a	1.98 ± 0.22 a
Roots	PSL + IN	20	0.19 ± 0.04	3.27 ± 0.16 c	0.11 ± 0.03 b	14.97 ± 0.78 b
	PSL	20	0.72 ± 0.23	5.76 ± 0.82 c	0.47 ± 0.13 a	32.55 ± 3.14 a
	UL + IN	18	0.26 ± 0.05	15.05 ± 0.90 b	0.16 ± 0.03 b	17.19 ± 0.82 b
	UL	19	0.28 ± 0.07	18.62 ± 1.40 a	0.21 ± 0.05 b	27.40 ± 1.81 a

*Panel A from Michaelis-Menten curves fitted on results from solutions S1 to S9 (Ala-1-^*13*^C,^*15*^N at IN:ON ratios 10:1 to 500:1), on whole plant uptake per root dry weight. Panel B from solutions S7 to S18 (Ala-1-^*13*^C,^*15*^N or U-Ala-^*13*^C_3_,^*15*^N, with or without IN at IN:ON ratio 10:1), separately in shoots and roots. ^*13*^C and ^*15*^N enrichment were measured by BSIA. ^*13*^C enrichment was not significant in shoots of plants exposed to Ala-1-^*13*^C,^*15*^N, hence the empty section. The Michaelis–Menten curves were fitted using the Ala concentration in the treatment solutions, not the total N concentration. Within one plant tissues (shoot or root), treatments with a same letter are not significantly different from one another according to the Wald test (α = 0.05, adjusted according to the Benjamini–Hocheberg procedure with Q = 0.05). When there are no letters, no significant differences were found. Parameters ± SE.*

**FIGURE 3:**
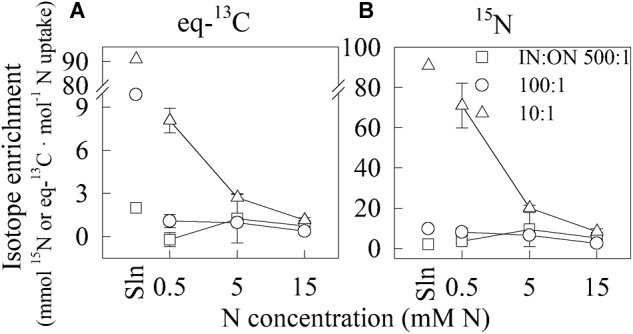
Effect of total N concentration and IN:ON ratio on eq-^13^C **(A)** and ^15^N **(B)** enrichment per total N uptake in cucumber seedlings, based on BSIA. Plants were exposed to three different concentrations of total N at three different IN:ON ratios (IN = NH_4_NO_3_-N and ON = Alanine-1-^13^C,^15^N-N): 500:1 (□), 100:1 (∘) and 10:1 (∆). Values are means ± SE. Points on the left (“Sln”) indicate the IN:ON ratio in the solution (mmol Ala ⋅ mol^-1^ total N). *n* = 4 for plants exposed to 0.5 and 5 mM N; *n* = 5 for the others.

When reducing the total N concentration from 15 to 0.5 mM N, the fraction of total N taken up from Ala increased 2.9- and 6.6-fold (on an eq-^13^C basis) for ratios 10:1 and 100:1, respectively (Figure 3A). At ratio 10:1, for which this increase was the steepest, the fraction increased from 8.35‰ in the 15 mM N solution to 68.38‰ in the 0.5 mM N solution on a ^15^N enrichment basis (Figure 3B).

**FIGURE 4:**
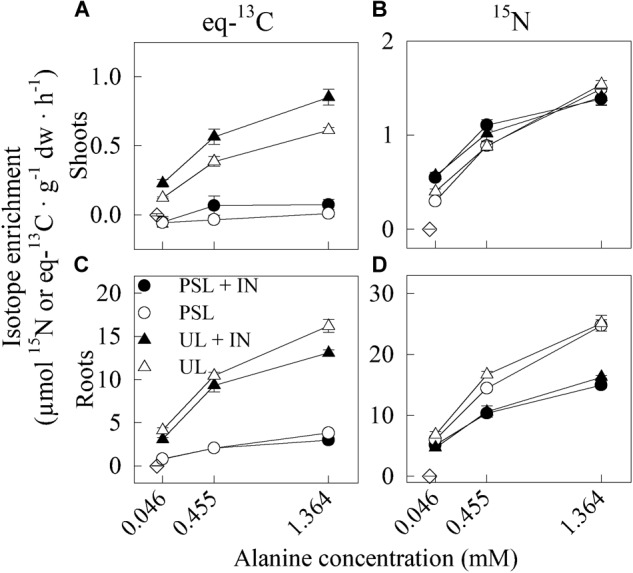
Influence of the presence or absence of IN (at a IN:ON ratio of 10:1) and of the different labeling of Ala on ^13^C-eq **(A,C)** and ^15^N **(B,D)** enrichment in shoots **(A,B)** and roots **(C,D)** of cucumber seedlings, based on BSIA. Plants were exposed to three different concentrations of Ala-1-^13^C,^15^N (∘) or U-Ala-^13^C_3_,^15^N (∆), with (black) or without (white) NH_4_NO_3_ (see solutions S7–S18 in Table 1). Values are means ± SE. Diamonds (◇) indicate the enrichment at 0 mM N. Treatments exposed to 0.455 mM Ala were also analyzed by CSIA (see Figure 5). *n* = 5 for all treatments, except for solutions containing 1.364 mM U-Ala-^13^C_3_,^15^N (S15 and 18), for which *n* = 4.

The maximum eq-^13^C uptake rate (V_max_) was ca. 4 times higher in plants exposed to solutions containing U-Ala-^13^C_3_,^15^N than to Ala-1-^13^C,^15^N (Figure 4C and Table 2B). This difference was not significant in shoots and roots when calculated from ^15^N enrichment. In absence of NH_4_NO_3_, the K_m_ and V_max_ for ^15^N enrichment were ca. twice as high as in presence of NH_4_NO_3_ in plants exposed to both types of labeled Ala. This difference was, however, not significant in plants exposed to Ala-1-^13^C,^15^N, when calculated from ^13^C enrichment.

**FIGURE 5:**
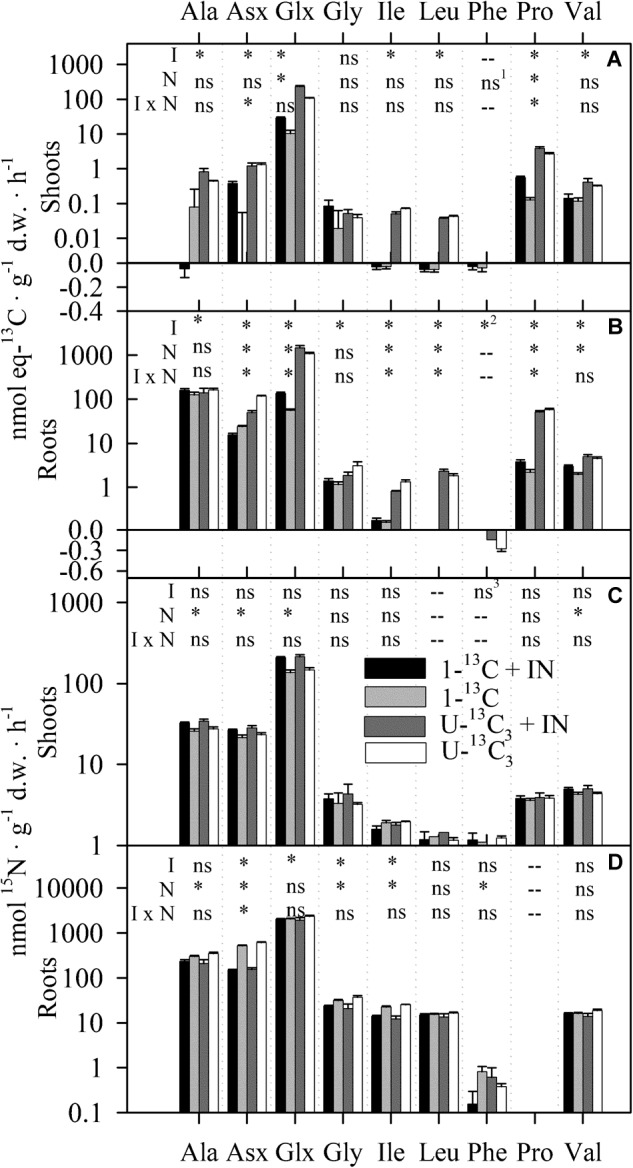
Compound specific isotope analysis (CSIA) by GC-C-IRMS of ^13^C **(A,B)** and ^15^N **(C,D)** assimilation in free amino acids in roots and shoots of cucumber seedlings exposed to 0.455 mM Ala-1-^13^C,^15^N or U-Ala-^13^C_3_,^15^N, with or without NH_4_NO_3_ at an IN:ON ratio of 10:1. The scale of the vertical axis is logarithmic over zero and linear below zero. The results of the ANOVA of the effects of the type of isotopologue (I), the presence of IN (N) and their interaction (I × N) are presented: ^∗^ indicates significance (α = 0.05) and ns means nonsignificant. – indicates that the effect could not be analyzed because of data below the limit of quantification: ^1^ANOVA comparing S8 (1-^13^C + IN) and S11 (1-^13^C) only; ^2^S11and S17 (U-^13^C_3_) only; ^3^only one data point for S11, thus one-way ANOVA comparing S8, S14 (U-^13^C_3_ + IN) and S17. See Table 1 for details on the content of the labeled solutions. *n* = 5 within each treatment.

The ^15^N and eq-^13^C assimilations in free amino acids are presented in Figure 5, along with the ANOVAs of the effects of isotopic labeling types, the presence of IN and their interaction (*P*-values presented in Supplementary Table 5). Glx (i.e., the sum of Gln and Glu) accounted for ca. 30 times the ^13^C transfer and 2.5 times the ^15^N transfer to the shoots relative to all other amino acids combined. In general, the type of labeled molecule (“I” effect in Figure 5) impacted the ^13^C assimilation in amino acids, whereas the presence or absence of IN (“N” effect in Figure 5) affected the ^15^N assimilation. Specifically, in roots, ^15^N assimilation in amino acids was 12% (Glx) to 274% (Asx, i.e., the sum of Asn and Asp) higher in plants exposed to Ala alone than in plants which were exposed to Ala with IN (Figure 5D). In shoots, all amino acids were enriched in ^15^N, with no difference between the two types of labeled Ala (Figure 5C). However, plants exposed to IN along with ON assimilated in their shoots up to 25, 23, and 50% more ^15^N as, respectively, free Ala, Asx, and Glx than plants exposed to ON only. The eq-^13^C assimilation was much higher for all amino acids in plants exposed to U-Ala-^13^C_3_,^15^N than to Ala-1-^13^C,^15^N, except for glycine in the shoots; this difference, although significant, was only 5.7% for Ala in the roots (Figures 5A,B). Glu and Gln (Glx) were the amino acids in which ^13^C was most assimilated in the roots exposed to U-Ala-^13^C_3_,^15^N, accounting for 85 and 76% (respectively with and without IN) of the ^13^C in all free amino acids. In roots exposed to Ala-1-^13^C,^15^N, Ala accounted for 50 and 59% (respectively with and without IN) of the ^13^C present in all free amino acids. Shoots of plants exposed to Ala-1-^13^C,^15^N were significantly depleted in ^13^C in Leu (Figure 5A; Ile was not significantly depleted according to Dunnett test, data not shown).

A comparison of the eq-^13^C and ^15^N uptake values reported by CSIA or BSIA, after exposure to Ala-1-^13^C,^15^N or U-Ala-^13^C_3_,^15^N alone (solutions S11 and S17 in Table 1), is presented in Table 3. Exposing plants to Ala-1-^13^C,^15^N instead of U-Ala-^13^C_3_,^15^N resulted in a fivefold decrease in the eq-^13^C uptake monitored by BSIA in the roots, and the ^13^C enrichment in the shoots was below detection limits. Calculating the eq-^13^C and ^15^N assimilation in free Ala by CSIA resulted in uptake rates 16–64 times lower than when monitored by BSIA. When summing up the eq-^13^C or ^15^N assimilation in all free amino acids, the total enrichment accounted for 10–29% of the enrichment monitored by BSIA, suggesting that 10–29% of the eq-^13^C and ^15^N taken up as intact Ala is present as free amino acids in roots and shoots of the plant, 2 h after the beginning of exposure to the solution.

**Table 3:** Comparison of results from six different analytical methods for estimation of intact Ala uptake.

		CSIA				BSIA	
	Plant Part	Ala-1-^13^C,^15^N (S11)		U-Ala-^13^C_3_,^15^N (S17)		Ala-1-^13^C, ^15^N (S11)	U-Ala-^13^C_3_, ^15^N (S17)
Unit		Free Ala	All free AA	Free Ala	All free AA		
μmol ^15^N ⋅ g^-1^ dw ⋅ h^-1^	Shoots	0.026 ± 0.004	0.200 ± 0.035	0.028 ± 0.004	0.214 ± 0.033	0.890 ± 0.106	0.883 ± 0.102
	Roots	0.302 ± 0.044	3.00 ± 0.23	0.356 ± 0.054	3.48 ± 0.27	14.46 ± 0.54	16.73 ± 1.13
μmol eq-^13^C ⋅ g^-1^ dw ⋅ h^-1^	Shoots	8.0E-5 ± 4.02E-4	0.011 ± 0.005	4.54E-4 ± 4.3E-5	0.112 ± 0.016	–0.036 ± 0.030	0.387 ± 0.080
	Roots	0.128 ± 0.037	0.214 ± 0.034	0.164 ± 0.032	1.45 ± 0.20	2.07 ± 0.07	10.46 ± 0.90

*^*15*^N and eq-^*13*^C uptake rates were monitored by the use of CSIA or BSIA, after exposing cucumber seedlings to solutions containing dual-labeled Ala. Results presented are from plants exposed to solutions S11 (0.455 mM Ala-1-^*13*^C,^*15*^N without IN; n = 5) and S17 (0.455 mM U-Ala-^*13*^C_3_,^*15*^N without IN; n = 5). See also Figure 4 for BSIA and Figure 5 for CSIA results. Within the results of CSIA, the enrichment of free Ala only and the sum of the enrichment in all free amino acids are both presented. Gray values: Shoots of plants exposed to solutions S11 were not significantly enriched in ^*13*^C (see Figure 1). Free Ala-treatment was also not significantly enriched in ^*13*^C in shoots of the same plant. Values are means of 5 samples ± SD.*

## Discussion

### Alanine Uptake

Our results showed that cucumber seedlings exposed to solutions containing IN and Alanine N can take up part of their N as intact Ala at concentrations typically used in organic greenhouse horticulture. This is in agreement with previous studies on plant uptake of amino acids in presence of inorganic N (Ge et al., 2009; Gioseffi et al., 2012; Gruffman et al., 2014; Czaban et al., 2016).

In soil, IN and ON occur at different ratios, e.g., during the season or after application of fertilizer. What is unique in the present study is the investigation of this aspect, specifically of different ratios of IN and ON and different concentrations relevant to organic greenhouse horticulture of cucumber. The plants were able to increase their total ON uptake when exposed to a higher total N concentration and Ala concentration within each IN:ON ratio in presence of IN (Figure 2). In accordance with kinetic studies of amino acid uptake (Soldal and Nissen, 1978; Gruffman et al., 2014), the increase in uptake was not linear but followed Michaelis-Menten kinetics (Figure 2 and Table 2). The uptake of Ala was highest at the smallest ratio (10:1) which correlates to the smallest proportion of IN to ON but also to the largest proportions of Ala (Figure 2). This confirms the capacity of plants to benefit from increased ON availability. Plants possess both high and low affinity amino acid transporters, with K_m_ values, respectively, <50 μM and >500 μM (Henry and Jefferies, 2003; Rentsch et al., 2007; Tegeder and Rentsch, 2010). Low-affinity transporters (high K_m_) usually provide passive, high rate transport, but only manage to take up amino acids at a high concentration. High-affinity transporters (low K_m_) rely on active, low rate transport, but achieve uptake at lower substrate concentration. The K_m_ and V_max_ values reported here are based on the net uptake, spanning the action of multiple types of amino acid transporters. A higher K_m_ suggests that a higher proportion of the uptake is the result of the action of low affinity transporters. Accordingly, the K_m_ and V_max_ values observed at a IN:ON ratio of 10:1 were higher than at ratio 100:1 (not significantly different from the 500:1 ratio, which had higher standard errors). This indicates that the low-affinity amino acid transporters can take advantage of the higher presence of ON relative to IN.

In absence of NH_4_NO_3_, the roots exposed to Ala took up more N as Ala than in presence of both NH_4_NO_3_ and Ala. Removing altogether the IN from the solution affected Ala uptake in the same direction as decreasing the IN:ON ratio; the ensuing increase in Ala-N uptake can thus be attributable to the need for the plant to find alternative N sources. This hypothesis was also confirmed in white clover (Czaban et al., 2016). Moreover, some species adapted to low IN environments (Gruffman et al., 2014), or even agricultural plants such as wheat (Gioseffi et al., 2012) can have their ON uptake unaffected by the presence or absence of nitrate and rely on organic N sources for a significant part of their N budget. In our experiment, the uptake of Ala measured in roots was in agreement with this hypothesis, though higher quantities of C and N were transferred from labeled Ala to the shoots in presence of NH_4_NO_3_ than when exposed to Ala alone (Figure 4; ANOVA in Supplementary Table 4). There were, however, no significant differences in K_m_ and V_max_ values. This is in contradiction with the results for white clover reported by Czaban et al. (2016), who found a decrease in Arg transfer to the shoot in presence of IN. The recent proposition by Franklin et al. (2017) that the C assimilated from organic N sources increases N use efficiency (NUE) could help explaining our results: the C cost associated to the additional uptake of N from inorganic source may further stimulate the transfer of C from the roots to the shoots in order to contribute to N assimilation at the shoot level. Since the only C source available for uptake by the roots in our experiment was Ala, the ^15^N included in the molecule could have come along and stimulated a higher IN uptake. Besides, an increase in NUE due to a greater C availability can explain the much higher IN uptake observed at IN:ON ratios of 10:1 and 100:1 than at 500:1 (Figure 2C). Gruffman et al. (2014) also observed a slightly higher nitrate uptake in Scots pine seedlings pre-exposed to a high concentration of Arg and IN. Gioseffi et al. (2012), however, reported a decrease in nitrate uptake when exposing wheat plants to Gly along with nitrate, relative to nitrate alone; so did Thornton and Robinson (2005) after exposing ryegrass to a mixed nitrate – ammonium – glycine solution, relative to a nitrate-only solution, and Stoelken et al. (2010) after exposing European beech to a mixed nitrate – ammonium – Arg or Gln solution, relative to nitrate and ammonium only. In the four cases, however, the N concentration of the treatment solution was much lower (respectively 0.5, 3, 0.66, and 3.5 mM IN) than in our experiment (5 and 15 mM total N). The increased presence of C as Ala not only was beneficial, but a certain amount of it appeared essential to attain a high IN uptake rate. This effect encourages fertilization of crops with an organic source of N to complement highly concentrated mineral N fertilization (>5 mM N), such as encountered in conventional greenhouse fertigation.

In all studied solutions, the ratio of Ala-N uptake to total N uptake was always lower than the ratio of available Ala-N to total N in the solution (Figure 3). This finding supports the well-accepted assumption that roots have a preference for IN over amino acids in agricultural contexts (Okamoto and Okada, 2004; Schimel and Bennett, 2004; Nacry et al., 2013), even at ON concentrations representative of greenhouse organic horticulture. Indeed, at total N concentration of 15 mM N, only 0.4–0.9% of the total N that was taken up by seedlings came from Ala (Figure 3B). The highest proportion of Ala, 6.8%, was taken up at a IN:ON ratio of 10:1 and a total N concentration of 0.5 mM, which is, however, too low for horticulture application in greenhouse context.

Using a dual-labeled amino acid with only the carboxyl C as ^13^C for assessing its intact uptake rate had the intended purpose of reducing the biases occurring when relying on universally labeled amino acids, due to their pre- and post-uptake metabolism. The enrichment in eq-^13^C observed using U-Ala-^13^C_3_,^15^N was much higher than that found with Ala-1-^13^C,^15^N, even when accounting for the presence of three ^13^C atoms in the former compared to only one in the later (hence the use of eq-^13^C instead of ^13^C). The bias associated with universally labeled amino acids in the determination of intact amino acid uptake can result from many sources. First, mineralization of the labeled molecules can occur outside the roots in the rhizosphere, mainly by microorganisms (Schimel and Bennett, 2004), but also to a lesser extent by exoenzymes secreted in root exudates (Czaban et al., 2016). Second, post-uptake metabolism of the amino acid such as decarboxylation (Moran-Zuloaga et al., 2015) can reduce the isotopic enrichment. Moreover, no eq-^13^C enrichment was detected in shoots of plants exposed to Ala-1-^13^C,^15^N based on BSIA, whereas there was eq-^13^C transferred to the shoots of plants exposed to U-Ala-^13^C_3_,^15^N (Figure 1A). This further supports the occurrence of a rapid post-uptake metabolism of amino acids, mainly affecting the carboxyl-C (Moran-Zuloaga et al., 2015). It must, however, be considered that the natural abundance of ^13^C (1.07 at.% ^13^C in shoots of plants exposed to the control solution S0) was higher than for ^15^N (0.37 at.% ^15^N in S0), and that the concentration of C (39.47% C in S0) was higher than the concentration of N (4.06% in S0) in plants. This resulted in a larger dilution of ^13^C from labeled Ala in samples compared to ^15^N, making detection of ^13^C excess by BSIA harder than for ^15^N. Indeed, a small but significant eq-^13^C enrichment was monitored for free Asx, Glx, Pro, and Val in shoots of plants exposed to Ala-1-^13^C,^15^N (Figure 5A). In any case, albeit the various biases encountered in the calculation of the intact uptake rate, exposing the plant to UL alanine is still informative on the total uptake and assimilation of N and C provided by Ala.

### Alanine Assimilation

Plants possess an array of catabolic pathways for amino acids, which allow the reuse of amino acids and recycling of N (Hildebrandt et al., 2015; Yoneyama et al., 2016). The isotopic enrichment of each amino acid, obtained by CSIA, in combination with both position-specific and universally labeled Ala, provides insightful information on the fate of ^13^C and ^15^N along those pathways. It thus allows for a clear demonstration that, in addition to being taken up intact, amino acids can be assimilated into the plant’s metabolism.

Carbon and N from free Ala can be incorporated in the amino acid metabolism through pyruvate (C) and Glu (N), from the action of alanine aminotransferase (Hildebrandt et al., 2015). This same enzyme has previously been studied for the positive effect of its reverse reaction on N use efficiency (Ala production from pyruvate and Glu), in a context of mineral fertilization only (Good et al., 2007; Shrawat et al., 2008). Pyruvate is then integrated to acetyl-CoA, with the loss, as CO_2_, of the carboxyl-C (^13^C in our experiment) of the amino acid. Acetyl-CoA is integrated in the citrate cycle, and both C atoms originally from Ala can be integrated in Glu and Asp (Gaufichon et al., 2016), thus explaining the high eq-^13^C enrichment in those two amino acids after exposure to U-Ala-^13^C_3_,^15^N. Since the carboxyl-C of Ala is lost as CO_2_, it is not surprising that a very low eq-^13^C enrichment was observed in amino acids other than Ala in the plants exposed to Ala-1-^13^C,^15^N, in which only the carboxyl-C was ^13^C-labeled. The ^13^C assimilation in free amino acids other than Ala that was found even after exposure to Ala-1-^13^C,^15^N could be explained at least in part by the uptake of carbonate resulting from the decarboxylation of Ala outside the roots (Vuorinen et al., 1989; Sauheitl et al., 2009; Rasmussen et al., 2010): bicarbonate can be used in the carboxylation of phosphoenolpyruvate, resulting in the production of malate that can be integrated into the citrate cycle. Yet, in roots, free Ala had a similar eq-^13^C enrichment after exposure to both types of labeled Ala; it is a strong indicator that the ^13^C enrichment observed in free Ala came from intact uptake, both when exposing the plant to Ala-1-^13^C,^15^N and U-Ala-^13^C_3_,^15^N.

Glutamate and Glutamine are at the heart of IN assimilation through the GS/GOGAT pathway. Nitrate taken up by the roots is reduced to NO_2_^-^ by Nitrate reductase, to NH_4_^+^ by Nitrite reductase, included in Glutamine by Glutamine synthetase, then transaminated to Glutamate by Glutamate synthase (Xu et al., 2012). After that, N can be transferred to Ala also by the action of alanine aminotransferase (Good et al., 2007). The ^14^N from ammonium nitrate can thus dilute the ^15^N pool of Alanine (thus decreasing its δ^15^N), with little effect on its δ^13^C. This can explain how Ala in roots exposed to ON + IN was depleted in ^15^N relative to roots exposed to ON alone, while there was no effect of the presence of IN on δ^13^C (Figures 5B,D). Moreover, Glu and Gln (Glx) were by far the main free amino acids responsible for the transport to the shoots of the ^13^C and ^15^N taken up by the roots, suggesting rapid metabolism of Ala after its uptake. Glu and Gln being at the center of the N assimilation pathways, it is to be expected that a significant portion of the assimilated C and N would become part of them. Proline, a direct product of Glu, was also strongly enriched in ^13^C.

One unexpected result is the ^13^C depletion observed in Leu (also present, but not significant in Phe and Ile according to Dunnett test; data not shown) in shoots exposed to Ala-1-^13^C,^15^N. Leu is a down-product from pyruvate (Galili et al., 2016). Downstream in the metabolic pathway leading to Leu, the carboxyl-C from pyruvate is lost as CO_2_, and two C from Acetyl-CoA are included. The non-carboxyl C atoms from Ala-1-^13^C,^15^N (thus also in pyruvate produced from the catabolism of Ala), and the two C from Acetyl-CoA (that can also be coming from the two non-carboxyl C in pyruvate) could be depleted in ^13^C depending on the source material used for their production. This could explain the ^13^C depletion of Leu, although confirming this is outside the scope of this study.

### Comparison of BSIA With CSIA

Unsurprisingly, the rates of ^13^C and ^15^N assimilation in free amino acids determined by BSIA were much higher (between 16 and 64 times) than those observed using CSIA. This is in accordance with the results of Sauheitl et al. (2009), who found a 1.6–8 times higher uptake rate when using BSIA compared with CSIA after exposing plants to dual-labeled amino acids. The larger difference that we observed between BSIA and CSIA as compared to Sauheitl et al. (2009) could be due to the fact that we performed CSIA on free amino acids compared to their CSIA analysis of samples containing amino acids from proteins, or to our shorter exposure to labeled solution (2 h compared to 24 h for Sauheitl et al., 2009). Our analysis targeting only free amino acids and our short incubation time likely decreased the proportion of amino acids incorporated in proteins and could give a better snapshot of the rapid post-uptake metabolism of amino acids. However, considering the rapid post-uptake metabolism unraveled by CSIA, BSIA of plants exposed to a PSL amino acid appears more accurate in the estimation of intact Ala uptake rate.

## Conclusion

The use of CSIA and BSIA, in combination with PSL and universally labeled Alanine, provided unique information on the uptake and assimilation of labeled Ala in free amino acids in cucumber seedlings. Our results contribute to the increasing awareness that uptake of ON in the form of intact amino acids is possible and significant for the plant N budget. In the context of field organic horticulture, understanding the post-uptake metabolism of different amino acids will help predict and model the nutritive value of different organic N fertilizers for the plant. However, at high N concentration such as that used for greenhouse crops, amino acid uptake appears to have only a marginal role in the total crop N budget. Future research on ON uptake should be widened to different crops and agricultural practices, such as organic farming, since the preferred source of N varies from one species to another and, for a given species, among different nutritional contexts. A substantial achievement would be to quantify the global ON uptake in field conditions and adjust recommendations for N fertilization accordingly.

## Author Contributions

P-PD designed and performed the experiment, analyzed the data and wrote the first version of the article. SP and MD obtained the funding for the project. SP, MD, and SJ participated in the planning of the experiment. AB contributed to the analysis of amino acids. SP, MD, SJ, and AB helped in the interpretation of the results and revised the manuscript.

## Conflict of Interest Statement

The authors declare that the research was conducted in the absence of any commercial or financial relationships that could be construed as a potential conflict of interest.

## Abbreviations

BSIA: Bulk stable isotope analysis
CSIA: Compound specific isotope analysis
IN: Inorganic nitrogen
ON: Organic nitrogen
PSL: Position-specific labeling
UL: Universally labeled
