# Supplementary material for: Organic Nitrogen Uptake and Assimilation in Cucumis sativus Using Position-Specific Labeling and Compound-Specific Isotope Analysis
Source: Front Plant Sci. 2018 Nov 6;9:1596. doi: 10.3389/fpls.2018.01596 (PMC6232311; doi:10.3389/fpls.2018.01596)
Supplement: Supplementary file 1 [file Table_1.docx]

***Supplementary Material***

**Organic nitrogen uptake and assimilation in *Cucumis sativus* using position-specific labeling and compound-specific isotope analysis**

Pierre-Paul Dion^1*^, Sandra Jämtgård^2^, Annick Bertrand^3^, Steeve Pepin^4^ & Martine Dorais^1^

*Authors for correspondence: Pierre-Paul Dion ([pierre-paul.dion.1@ulaval.ca](mailto:pierre-paul.dion.1@ulaval.ca)) and Martine Dorais ([martine.dorais.1@ulaval.ca](mailto:martine.dorais.1@ulaval.ca))

Supplementary Figure 1. Concentration of free amino acids in shoots and roots of young cucumber plants, used for extrapolation of the concentration of free amino acids in samples analyzed by CSIA (see Supplementary Table 1). The plants were exposed to solutions containing NH_4_NO_3_ and Ala-1-^13^C_3_,^15^N varying in IN:ON ratio and total N concentration. S1 = IN:ON ratio 500:1, 0.5 mM total N; S5 = IN:ON ratio 100:1, 5 mM N; S7 = IN:ON ratio 10:1, 0.5 mM N; S9 = IN:ON ratio 10:1, 15 mM N; S10 = Ala alone, 0.046 mM N; S12 = Ala alone, 1.364 mM N. See Table 1 in main manuscript for the detailed composition of the solutions.

Supplementary Table 1. Correspondence between the samples subjected to CSIA and those from which the free amino acids were quantified in order to estimate the eq-^13^C and ^15^N uptake and assimilation rate (see Fig. 5 of the manuscript). The choices of correspondence are described below the Table and are based on results presented in Supplementary Figure 1. Where two solutions are indicated, the mean of the concentration observed in both treatments was used.

| Samples analyzed by CSIA | |  | Samples from which the free amino acid was quantified | | | | | | | | | | |
| --- | --- | --- | --- | --- | --- | --- | --- | --- | --- | --- | --- | --- | --- |
| Part | Labelled solution |  | Ala | Asn | Asp | Gln | Glu | Gly | Ile | Leu | Phe | Pro | Val |
| Shoots | S8 |  | S5 | S5 | S7 & 9 | S5 | S5 | S7 & 9 | S5 | S5 | S5 | S5 | S5 |
|  | S11 |  | S10 & 12 | S10 & 12 | S10 & 12 | S10 & 12 | S10 & 12 | S10 & 12 | S10 & 12 | S10 & 12 | S10 & 12 | S10 & 12 | S10 & 12 |
|  | S14 |  | S5 | S5 | S7 & 9 | S5 | S5 | S7 & 9 | S5 | S5 | S5 | S5 | S5 |
|  | S17 |  | S10 & 12 | S10 & 12 | S10 & 12 | S10 & 12 | S10 & 12 | S10 & 12 | S10 & 12 | S10 & 12 | S10 & 12 | S10 & 12 | S10 & 12 |
| Roots | S8 |  | S7 & 9 | S5 | S7 & 9 | S5 | S7 & 9 | S5 | S7 & 9 | S5 | S7 & 9 | S5 | S5 |
|  | S11 |  | S10 & 12 | S10 & 12 | S10 & 12 | S10 & 12 | S10 & 12 | S10 & 12 | S10 & 12 | S10 & 12 | S10 & 12 | S10 & 12 | S10 & 12 |
|  | S14 |  | S7 & 9 | S5 | S7 & 9 | S5 | S7 & 9 | S5 | S7 & 9 | S5 | S7 & 9 | S5 | S5 |
|  | S17 |  | S10 & 12 | S10 & 12 | S10 & 12 | S10 & 12 | S10 & 12 | S10 & 12 | S10 & 12 | S10 & 12 | S10 & 12 | S10 & 12 | S10 & 12 |

In shoots, and in some cases in roots, the concentration of free amino acids appeared to be influenced mostly by the abundance of total N (in presence of IN). Therefore, the concentration of free amino acids in plants exposed to S5 (5 mM N, at IN:ON ratio 100:1) was considered as representative of the free amino acid concentration in plants exposed to S8 and S14 (Ala-1-^13^C,^15^N and U-Ala-^13^C_3,_^15^N with IN at IN:ON ratio 10:1, 5 mM N total N) for all free amino acids except Gly and Asp in the shoots and Ala, Asp, Glu, Ile and Phe in the roots. In these last cases, the abundance of the amino acid appeared to be influenced both by the total N concentration and the IN:ON ratio. The mean of the concentration of free amino acids in plants exposed to S7 and S9 (0.5 and 15 mM N, at IN:ON ratio 10:1) was considered as representative of the free amino acid concentration in plants exposed to S8 and S14 for Gly and Asp in the shoots and Ala, Asp, Glu, Ile and Phe in the roots.

For all the amino acids, both in roots and shoots, the mean of the concentration of free amino acids in plants exposed to S10 and S12 (0.046 and 1.364 mM Ala alone) was considered as representative of the free amino acid concentration in plants exposed to S11 and S17 (Ala-1-^13^C,^15^N and U-Ala-^13^C_3,_^15^N without IN, at 0.455 mM Ala).





Supplementary Figure 2. Regression analysis of the ^13^C excess against the ^15^N excess in shoots and roots for all treatments. See Supplementary Table 2 for the linear regression equations. “ns” = non-significant linear relationship (P > 0.05); * P < 0.05; *** P < 0.001. The statistical significance shown in the bottom-right corners refers to the overall linear relationship covering the three N levels, whereas those closer to data points refer to the linear relationship within each total N concentration.

|  |  | Alanine with NH_4_NO_3_ (IN:ON ratio) | | | | | | | | | | |  | Alanine only | | | | |
| --- | --- | --- | --- | --- | --- | --- | --- | --- | --- | --- | --- | --- | --- | --- | --- | --- | --- | --- |
|  | Isotope | Ala-1-^13^C,^15^N | | | | | | | |  | U-Ala-^13^C_3_,^15^N | |  | Ala-1-^13^C,^15^N | |  | U-Ala-^13^C_3_,^15^N | |
|  | Ratio | 500:1 | |  | 100:1 | |  | 10:1 | |  |  | |  | --- | |  | --- | |
|  | Part | Shoot | Root |  | Shoot | Root |  | Shoot | Root |  | Shoot | Root |  | Shoot | Root |  | Shoot | Root |
| All concen-trations | ß_1_ | 4.3E-04 | **0.211** |  | -0.076 | **0.231** |  | 0.110 | **0.207** |  | **2.214** | **2.423** |  | 0.048 | **0.163** |  | **1.284** | **1.972** |
|  | ß_0_ | -0.130 | **0.026** |  | -0.049 | **-0.015** |  | -0.144 | **-0.281** |  | **-1.131** | **-0.028** |  | -0.141 | **-0.467** |  | **-0.164** | **-2.420** |
|  | R^2^ | 1.1E-06 | **0.784** |  | 0.080 | **0.916** |  | 0.128 | **0.908** |  | **0.985** | **0.848** |  | 0.187 | **0.975** |  | **0.940** | **0.997** |
|  | P-val | 0.997 | **1.3E-04** |  | 0.373 | **1.0E-06** |  | 0.208 | **4.3E-08** |  | **2.4E-12** | **2.9E-06** |  | 0.107 | **8.6E-12** |  | **1.0E-08** | **1.9E-16** |
| 0.5 mM N | ß_1_ | -7.456 | -0.071 |  | -2.147 | 0.012 |  | 0.696 | 0.101 |  | 1.479 | **2.657** |  | **-0.881** | *0.152* |  | -0.468 | **1.782** |
|  | ß_0_ | 0.338 | 2.4E-04 |  | 0.275 | 0.229 |  | -0.826 | 0.619 |  | -0.317 | **-6.441** |  | **0.417** | *-0.250* |  | 1.118 | **0.442** |
|  | R^2^ | 0.798 | 3.3E-03 |  | 0.091 | 3.7E-04 |  | 0.454 | 0.383 |  | 0.376 | **0.848** |  | **0.864** | *0.707* |  | 0.347 | **0.965** |
|  | P-val | 0.107 | 0.942 |  | 0.699 | 0.981 |  | 0.326 | 0.266 |  | 0.272 | **0.026** |  | **0.022** | *0.074* |  | 0.296 | **2.8E-03** |
| 5 mM N | ß_1_ | 0.387 | -0.056 |  | -0.113 | 0.045 |  | 0.157 | -0.076 |  | **2.074** | -0.818 |  | -0.167 | -0.022 |  | 1.712 | **2.170** |
|  | ß_0_ | -0.222 | 0.732 |  | -0.019 | 1.557 |  | -0.217 | 5.720 |  | **-0.833** | 73.387 |  | 0.226 | 4.769 |  | -0.702 | **-9.822** |
|  | R^2^ | 0.152 | 0.077 |  | 0.132 | 0.034 |  | 0.015 | 0.401 |  | **0.984** | 0.115 |  | 0.342 | 0.027 |  | 0.530 | **0.840** |
|  | P-val | 0.610 | 0.722 |  | 0.637 | 0.816 |  | 0.847 | 0.251 |  | **8.4E-04** | 0.576 |  | 0.300 | 0.790 |  | 0.163 | **0.028** |
| 15 mM N | ß_1_ | *0.286* | 0.077 |  | -0.300 | -0.030 |  | -0.112 | -0.081 |  | **2.087** | **5.144** |  | 0.056 | *0.249* |  | 0.118 | **1.725** |
|  | ß_0_ | *-0.289* | 0.619 |  | 0.243 | 3.183 |  | 0.458 | 8.457 |  | **-0.756** | **-89.298** |  | -0.147 | *-4.700* |  | 3.325 | **10.644** |
|  | R^2^ | *0.864* | 0.472 |  | 0.040 | 7.7E-03 |  | 0.049 | 0.497 |  | **0.947** | **0.957** |  | 0.037 | *0.752* |  | 9.1E-03 | **0.979** |
|  | P-val | *0.071* | 0.313 |  | 0.800 | 0.912 |  | 0.720 | 0.184 |  | **0.027** | **0.022** |  | 0.756 | *0.057* |  | 0.905 | **0.011** |

Supplementary Table 2. Linear regression analysis of the ^13^C excess against the ^15^N excess in shoots and roots for all treatments. Equations follow the form ^13^C_excess_ = ß_1_ · ^15^N_excess_ + ß_0._ See also Supplementary Figure 2. Bold values are significant at α = 0.05; italic values are non-significant tendencies, at 0.1 > P > 0.05.

Supplementary Table 3. Two-way ANOVAs of the effects of N concentration, IN:ON ratio and their interaction on eq-^13^C and ^15^N enrichment (A and B, based on CSIA and labelled solutions S1 to S9) and on IN uptake (C, based on solution depletion). Uptake rates are expressed either on a per gram basis (A; C) or on a per total N uptake basis (B; whole plant). Values are presented in Figs. 2 and 3.

| A |  | µmol ^13^C · g^-1^ dw · h^-1^ | | | |  | µmol ^15^N · g^-1^ dw · h^-1^ | | | |
| --- | --- | --- | --- | --- | --- | --- | --- | --- | --- | --- |
| Plant part | Effect | Num df | Den df^†^ | *F* | *P* |  | Num df | Den df^†^ | *F* | *P* |
| Whole plant uptake per root d.w. | Conc. | 2 | 26.5 | 425.46 | **< 0.001** |  | 2 | 26.8 | 357.2 | **< 0.001** |
|  | Ratio | 2 | 25.9 | 769.78 | **< 0.001** |  | 2 | 26.3 | 853.6 | **< 0.001** |
|  | C × R | 4 | 25.8 | 56.94 | **< 0.001** |  | 4 | 26.1 | 10.1 | **< 0.001** |
|  |  |  | | | |  |  | | | |
| B |  | mmol ^13^C · mol^-1^ N_tot_ uptake | | | |  | mmol ^15^N · mol^-1^ N_tot_ uptake | | | |
| Whole plant | Conc. | 2 | 26.4 | 4.74 | **0.017** |  | 2 | 26.5 | 6.43 | **0.005** |
|  | Ratio | 2 | 25.1 | 21.90 | **< 0.001** |  | 2 | 25.3 | 26.17 | **< 0.001** |
|  | C × R | 4 | 24.7 | 3.34 | **0.026** |  | 4 | 25.1 | 2.97 | **0.039** |
|  |  |  |  |  |  |  |  |  |  |  |
| C |  | IN uptake (mmol IN · g^-1^ root dw · h^-1^) | | | | |  | | | |
| Whole plant uptake per root d.w. | Conc. | 2 | 25.2 | 56.7 | **< 0.001** |  |  |  |  |  |
|  | Ratio | 2 | 24.7 | 21.1 | **< 0.001** |  |  |  |  |  |
|  | C × R | 4 | 24.6 | 2.98 | **0.039** |  |  |  |  |  |

† Degrees of freedom at denominator were calculated with the Satterthwaite approximation (Kuznetsova et al., 2017).

Supplementary Table 4. Three-way ANOVA of the effects of the type of isotope labelling, the Alanine concentration and the presence of inorganic N (N_min_) on eq-^13^C or ^15^N enrichment in shoots and roots (labelled solutions S7 to S18). Data are presented in Fig. 4 of the manuscript.

|  |  | eq-^13^C | | | |  | ^15^N | | | |
| --- | --- | --- | --- | --- | --- | --- | --- | --- | --- | --- |
| Plant part | Effect | Num df | Den df^†^ | *F* | *P* |  | Num df | Den df^†^ | *F* | *P* |
| Shoots | Isotope* |  |  |  |  |  | 1 | 41.4 | 0.24 | 0.628 |
|  | Conc. | 2 | 22 | 105.47 | **< 0.001** |  | 2 | 41.4 | 343.93 | **< 0.001** |
|  | N_min_ | 1 | 22 | 31.69 | **< 0.001** |  | 1 | 41.1 | 8.47 | **0.006** |
|  | I × C |  |  |  |  |  | 2 | 41.3 | 1.20 | 0.312 |
|  | I × N |  |  |  |  |  | 1 | 41.1 | 1.13 | 0.294 |
|  | C × N | 2 | 22 | 1.47 | 0.251 |  | 2 | 41.1 | 11.55 | **< 0.001** |
|  | I × C × N |  |  |  |  |  | 2 | 41.1 | 0.09 | 0.912 |
| Roots | Isotope | 1 | 42.5 | 1616.48 | **< 0.001** |  | 1 | 46 | 4.18 | **0.047** |
|  | Conc. | 2 | 42.5 | 508.01 | **< 0.001** |  | 2 | 46 | 525.42 | **< 0.001** |
|  | N_min_ | 1 | 42.2 | 12.53 | **< 0.001** |  | 1 | 46 | 211.33 | **< 0.001** |
|  | I × C | 2 | 42.5 | 0.54 | 0.584 |  | 2 | 46 | 0.81 | 0.450 |
|  | I × N | 1 | 42.2 | 4.16 | **0.048** |  | 1 | 46 | 1.13 | 0.293 |
|  | C × N | 2 | 42.2 | 1.52 | 0.231 |  | 2 | 46 | 37.14 | **< 0.001** |
|  | I × C × N | 2 | 42.2 | 2.05 | 0.141 |  | 2 | 46 | 1.45 | 0.245 |

* ^13^C data in the shoots were included in the statistical analysis only for the treatments with U-Ala-^13^C_3_,^15^N, because there was no ^13^C enrichment monitored in the shoots for Ala-1-^13^C,^15^N (Fig. 1 in the manuscript).

^†^ Degrees of freedom at denominator were estimated with the Satterthwaite approximation (Kuznetsova et al., 2017).

Supplementary Table 5. P-values of the ANOVA on the effects of the isotope labelling type, mineral N presence/absence and their interaction (I × N) on the ^13^C and ^15^N enrichment of amino acids and their ratio. ^13^C enrichment is expressed as eq-^13^C to account for the ^13^C enrichment being three times higher in U-Ala-^13^C_3_,^15^N than in Ala-1-^13^C,^15^N. See Fig. 5 in the manuscript for the means. A gray field indicates that the amino acid was below limit of quantification.

|  |  |  | P-Values | | | | | | | | |
| --- | --- | --- | --- | --- | --- | --- | --- | --- | --- | --- | --- |
| Unit | Plant part | Effect | Ala | Asx | Glx | Gly | Ile | Leu | Phe | Pro | Val |
| mmol eq-^13^C-AA · mol^-1^ AA | Shoot | Isotope | **0.001** | **<0.001** | **<0.001** | 0.911 | **<0.001** | **<0.001** |  | **<0.001** | **<0.001** |
|  |  | Nmin | 0.845 | 0.807 | **<0.001** | 0.208 | *0.055* | *0.094* | 0.555* | **<0.001** | 0.942 |
|  |  | I × N | 0.191 | **0.003** | 0.243 | 0.469 | *0.075* | 0.126 |  | **<0.001** | 0.316 |
|  | Root | Isotope | **0.040** | **<0.001** | **<0.001** | **0.001** | **<0.001** | **<0.001** | **0.002§** | **<0.001** | **<0.001** |
|  |  | Nmin | *0.059* | **<0.001** | **<0.001** | 0.831 | **0.004** | **0.043** |  | **0.024** | **0.003** |
|  |  | I × N | 0.393 | **<0.001** | **0.003** | 0.177 | **0.001** | **0.027** |  | **0.009** | **0.588** |
| mmol eq-^15^N-AA · mol^-1^ AA | Shoot | Isotope | 0.338 | 0.255 | 0.477 | 0.786 | 0.327 |  | 0.128† | 0.905 | 0.402 |
|  |  | Nmin | **0.002** | **0.005** | **<0.001** | 0.257 | *0.066* |  |  | 0.854 | **0.007** |
|  |  | I × N | 0.986 | 0.884 | 0.779 | 0.688 | 0.564 |  |  | 0.339 | 0.576 |
|  | Root | Isotope | *0.058* | **0.001** | **0.011** | **0.003** | **0.039** | 0.123 | 0.958 |  | 0.182 |
|  |  | Nmin | **0.001** | **<0.001** | 0.364 | **<0.001** | **<0.001** | 0.339 | **0.045** |  | 0.161 |
|  |  | I × N | 0.362 | **0.016** | 0.518 | 0.519 | *0.059* | 0.893 | 0.149 |  | 0.251 |

*Phe in shoots (^13^C): ANOVA comparing S8 and S11 only (Ala-1-^13^C,^15^N, with and without IN)

§Phe in roots (^13^C): ANOVA on S11 and S17 only (isotope effect within treatments without IN)

†Phe in shoots (^15^N): Only 1 data point for S11 (Ala-1-^13^C,^15^N without IN) - ANOVA comparing the 3 other treatments
